# Network Meta-Analysis of Erlotinib, Gefitinib, Afatinib and Icotinib in Patients with Advanced Non-Small-Cell Lung Cancer Harboring EGFR Mutations

**DOI:** 10.1371/journal.pone.0085245

**Published:** 2014-02-12

**Authors:** Wenhua Liang, Xuan Wu, Wenfeng Fang, Yuanyuan Zhao, Yunpeng Yang, Zhihuang Hu, Cong Xue, Jing Zhang, Jianwei Zhang, Yuxiang Ma, Ting Zhou, Yue Yan, Xue Hou, Tao Qin, Xiaoxiao Dinglin, Ying Tian, Peiyu Huang, Yan Huang, Hongyun Zhao, Li Zhang

**Affiliations:** State Key Laboratory of Oncology in South China, Collaborative Innovation Center for Cancer Medicine, Sun Yat-sen University Cancer Center, Guangzhou, Guangdong, China; University of Nebraska Medical Center, United States of America

## Abstract

**Background:**

Several EGFR-tyrosine kinase inhibitors (EGFR-TKIs) including erlotinib, gefitinib, afatinib and icotinib are currently available as treatment for patients with advanced non-small-cell lung cancer (NSCLC) who harbor EGFR mutations. However, no head to head trials between these TKIs in mutated populations have been reported, which provides room for indirect and integrated comparisons.

**Methods:**

We searched electronic databases for eligible literatures. Pooled data on objective response rate (ORR), progression free survival (PFS), overall survival (OS) were calculated. Appropriate networks for different outcomes were established to incorporate all evidences. Multiple-treatments comparisons (MTCs) based on Bayesian network integrated the efficacy and specific toxicities of all included treatments.

**Results:**

Twelve phase III RCTs that investigated EGFR-TKIs involving 1821 participants with EGFR mutation were included. For mutant patients, the weighted pooled ORR and 1-year PFS of EGFR-TKIs were significant superior to that of standard chemotherapy (ORR: 66.6% vs. 30.9%, OR 5.46, 95%CI 3.59 to 8.30, *P*<0.00001; 1-year PFS: 42.9% vs. 9.7%, OR 7.83, 95%CI 4.50 to 13.61; *P*<0.00001) through direct meta-analysis. In the network meta-analyses, no statistically significant differences in efficacy were found between these four TKIs with respect to all outcome measures. Trend analyses of rank probabilities revealed that the cumulative probabilities of being the most efficacious treatments were (ORR, 1-year PFS, 1-year OS, 2-year OS): erlotinib (51%, 38%, 14%, 19%), gefitinib (1%, 6%, 5%, 16%), afatinib (29%, 27%, 30%, 27%) and icotinib (19%, 29%, NA, NA), respectively. However, afatinib and erlotinib showed significant severer rash and diarrhea compared with gefitinib and icotinib.

**Conclusions:**

The current study indicated that erlotinib, gefitinib, afatinib and icotinib shared equivalent efficacy but presented different efficacy-toxicity pattern for EGFR-mutated patients. Erlotinib and afatinib revealed potentially better efficacy but significant higher toxicities compared with gefitinib and icotinib.

## Introduction

Lung cancer is the leading cause of cancer-related mortality worldwide, with about 85% patients suffering from non-small cell lung cancer (NSCLC) [1]. At diagnosis, more than 80% of NSCLC cases are in advanced stage (IIIB or IV) for which systemic chemotherapy remains the standard care but provides marginal improvement in survival [2]. Epidermal growth factor receptor (EGFR)-dependent pathway, which is activated in more than half of patients with NSCLC, plays an important role in the development and progression of epithelial cells [3]. Small-molecule EGFR-tyrosine kinase inhibitors (TKIs) could competitively block the EGFR-dependent pathway [4]. In the last decade, a series of RCTs have confirmed the non-inferior efficacy and relatively low toxicity of erlotinib and gefitinib in treatment naïve or previously treated NSCLC patients compared with the standard chemotherapy [5]–[13]. Meanwhile, pre-planned or post-study biomarker analyses indicated that the presence of EGFR mutation, which mainly refers to deletions in exon 19 or the L858R substitution in exon 21, was the strongest predictor of efficacy for EGFR-TKIs. Thus, erlotinib and gefitinib have been included in NCCN guideline since 2010 as first-line treatment option for advanced NSCLC patients who harbor EGFR mutation [14]. Recently, two novel small molecule EGFR-TKIs were developed. Icotinib is a Chinese indigenous novel EGFR-TKI which has been approved by SFDA for second-line settings based on a large phase III RCT [15]. Afatinib is considered as a second-generation TKI that binds irreversibly to EGFR as well as receptors carrying the T790M mutation [16]. A phase II single arm study presented afatinib in NSCLC with EGFR activating mutations [17] and the efficacy of afatinib was compared with chemotherapy or erlotinib in a series of phase III RCTs named LUX-Lung [18]. Nevertheless, the relative effects of any of these TKIs compared with another in mutated patients remained unclear due to lack of evidence from head-to-head RCTs.

Network meta-analysis, also known as multiple-treatments comparison, enables us to synthesize data from both direct (within-trial comparisons) and indirect comparisons (inter-trial treatment comparisons through a common comparator treatment) of diverse regimens [19]. In addition, the Bayesian approach enables us to estimate the rank probability that, each of the treatments is the best, the second best, etc [20]. It is highly suggested that investigators should consider all potentially relevant data when comparing treatments and MTC is consistent with the true situation when adopting a wide network of studies that are chosen appropriately [21]. Thus, in the current study, we sought to provide some useful information about comparison between these four agents through integrating and indirect methods, expecting this message will be helpful for physicians and patients in decision-making.

## Methods

### Search Strategy

We searched PubMed, EMBASE and the Central Registry of Controlled Trials of the Cochrane Library using the combination of the search terms “non-small cell lung cancer”, “epidermal growth factor” OR EGFR, AND mutation within the restriction limit of “randomized controlled trial” (the deadline was March 2013). To identify updated outcomes from included trials or unpublished trials that had presented analyzed data, we also reviewed abstract books and presentations of major recent meetings of American Society of Clinical Oncology (ASCO), European Society for Medical Oncology (ESMO) and World Conference on Lung Cancer in 2008–2012. Finally, the reference lists of the included studies were reviewed as a supplement. No language limits were applied.

### Eligibility and Exclusion Criteria

The eligible studies should be phase III RCTs that compared one TKI (including erlotinib, gefitinib, afatinib and icotinib) to another or to standard chemotherapy as first-line or second-line treatments in patients with advanced NSCLC that presents activating EGFR mutations. Since the dominant histological type of patients with EGFR mutation was nonsquamous carcinoma in which pemetrexed were proved to yield superior efficacy compared with other third-generation chemotherapy agents, we also included studies that compared pemetrexed-based regimen with pemetrexed-free regimen in order to optimize the network. Notably, advanced NSCLC was defined as stage III or IV disease that was not feasible to surgical treatment or radiotherapy. Phase III RCTs were defined as studies with a power greater than 0.80 to detect a difference in survival. EGFR mutations mainly referred to deletions in exon 19 or the L858R substitution in exon 21. Standard chemotherapy was defined as platinum-based third generation doublets for first-line treatments or pemetrxed/doctaxel for second-line treatments. In cases of overlap reports, we included only the latest results. Studies failed to meet the inclusion criteria will be excluded.

### Quality Assessment and Data Extraction

The data collection and assessment of methodological quality follows the QUORUM and the Cochrane Collaboration guidelines (http://www.cochrane.de). The data on major clinical features, overall survival (OS), progression free survival (PFS), objective response rate (ORR) and adverse events (rash, grade 3–4 rash, diarrhea, grade 3–4 diarrhea) were extracted by two investigators (LW and WX) independently. Figures were electronically digitized and Kaplan-Meier curves were downloaded by appropriate software (Engauge Digitizer, ver 2.12, Mark Mitchell, 2002, free software down loaded from http://sourceforge.net). We rated the quality of each eligible study with JADAD score [22]. Discrepancies were discussed by the two investigators to reach consensus.

### Statistical Analyses

First, we conducted pair-wise meta-analyses with a random-effects model to synthesize studies comparing the same pair of treatments. The results were reported as pooled ORs with the corresponding 95% confidence interval (CI). Statistical heterogeneity across studies was assessed with a forest plot and the inconsistency statistic (I^2^). Statistical significance was considered at P<0.05. All calculations were performed using REVIEW MANAGER (version 5.0 for Windows; the Cochrane Collaboration, Oxford, UK).

Second, we built a random-effects network within a Bayesian framework using Markov chain Monte Carlo methods in ADDIS 1.15 (Drugis.org) [23]. We networked the translated binary outcomes of survival analysis and binary outcomes of ORR within studies and specified the relations among the ORs across studies making different comparisons as previously reported [24]. This method combined direct and indirect evidence for any given pair of treatments. We used P<0.05 and 95% CIs beyond the null value to assess significance.

We also estimated the probability that each of the treatment was the best regimen, the second best, the third best, and so on, by calculating the OR for each drug compared with an arbitrary common control group, and counting the proportion of iterations of the Markov chain in which each drug had the highest OR, the second highest, and so on. We ranked treatments in terms of efficacy and acceptability with the same methods.

A variance calculation and a node-splitting analysis provided by the software ADDIS 1.15 were applied to evaluate the inconsistency within the network meta-analysis. If the difference between random effects variance and inconsistency variance was large or a P<0.05 of disagreement between direct and indirect evidence was met, significant inconsistency was indicated. According to the quantitative estimation, we could adjust the study inclusion and ultimately obtain an ideal network with consistency.

## Results

### Eligible studies

**Figure 1:**
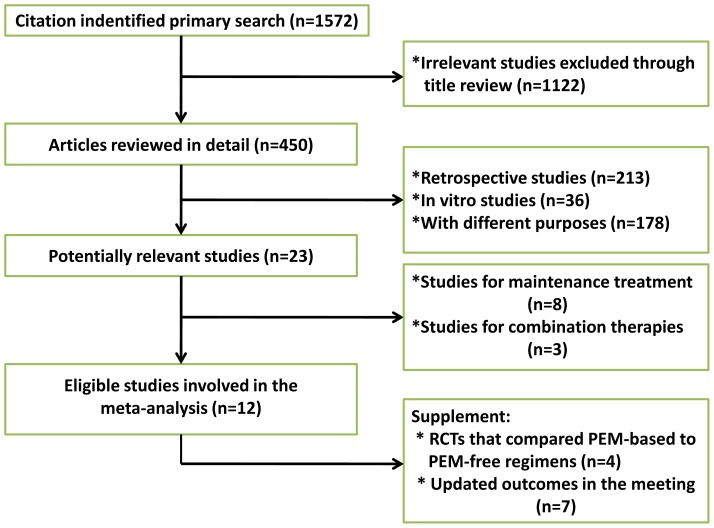
Profile summarizing the trial flow.

We identified 1572 records according to the search strategy and finally included 12 phase III RCTs that compared elotinib, gefitinib, icotinib, afatinib or chemotherapy in chemo-naïve or previously treated advanced NSCLC patients [5]–[13], [15], [25], [26]. Since LUX-lung 3 compared afatinib to pemetrexed in combination with cisplatin while the other studies compared TKIs to traditional regimens without pemetrexed, we included four RCTs that compared pemetrexed-based to pemetrexed-free regimens in predominantly nonsquamous carcinoma patients as a supplement to optimize the network [27]–[30]. Figure 1 summarized the flow chart. A total of 1821 patients were involved, among which 1066 patients received targeted drugs. First-SIGNAL [6], NEJ002 [7], WJTOG 3405 [8], OPTIMAL [11], EUTRAC [12] enrolled EGFR-mutated population only whereas the other included studies provided data from patients with mutation as pre-planned subgroup analyses or retrospective biomarker analyses. IPASS [5], first-SIGNAL [6], NEJ002 [7], WJTOG3405 [8], OPTIMAL [11], ICOGEN [15] and LUX-lung6 [26] predominantly enrolled Asian patients whereas EUTRAC [12] and TITAN [13] predominantly enrolled Caucasian. LUX-lung3 [25] is a global study that included both Asian and Caucasian. The majority of the included studies investigated TKIs as first-line treatment except for INTEREST [9], V 15–32 [10], TITAN [13] and ICOEGN [15] which investigated second-line treatments. Table 1 summarized the characteristics of all involved studies.

**Table 1:** Characteristics of included studies regarding TKIs.

Studies	TKI	Control	Year	Sample size	Patients status	EGFR Pts analyzed
**IPASS^5^**	Gefitinib	TC	2009	1217	CT-naive	261
**First-SIGNAL^6^**	Gefitinib	GP	2012	309	CT-naive	42
**NEJ002^7^**	Gefitinib	TC	2010	228	CT-naive	228
**WJTOG 3405^8^**	Gefitinib	DP	2010	172	CT-naive	117
**INTEREST^9^**	Gefitinib	DOC	2008	1466	Previously treated	38
**V 15–32^10^**	Gefitinib	DOC	2008	490	Previously treated	20
**OPTIMAL^11^**	Erlotinib	GC	2011	165	CT-naive	154
**EUTRAC^12^**	Erlotinib	CT	2012	174	CT-naive	173
**TITAN^13^**	Erlotinib	PEM/DOC	2012	424	Previously treated	11
**LUX-lung 3^25^**	Afatinib	AP	2013	345	CT-naive	345
**LUX-lung 6^26^**	Afatinib	GP	2013	364	CT-naive	364
**ICOGEN^15^**	Icotinib	Geftinib	2012	399	Previously treated	68

TKI, tyrosine kinase inhibitors; TC, carboplatin plus palitaxel; GP, cisplatin plus gemcitabine; DP, cisplatin plus docetaxel; DOC, docetaxel; GC, carboplatin plus gemcitabine; CT, chemotherapy (not specific); PEM, pemetrexed; AP, cisplatin plus pemetrexed.

### Pooled Weighted Outcomes and Direct Meta Analysis

**Figure 2:**
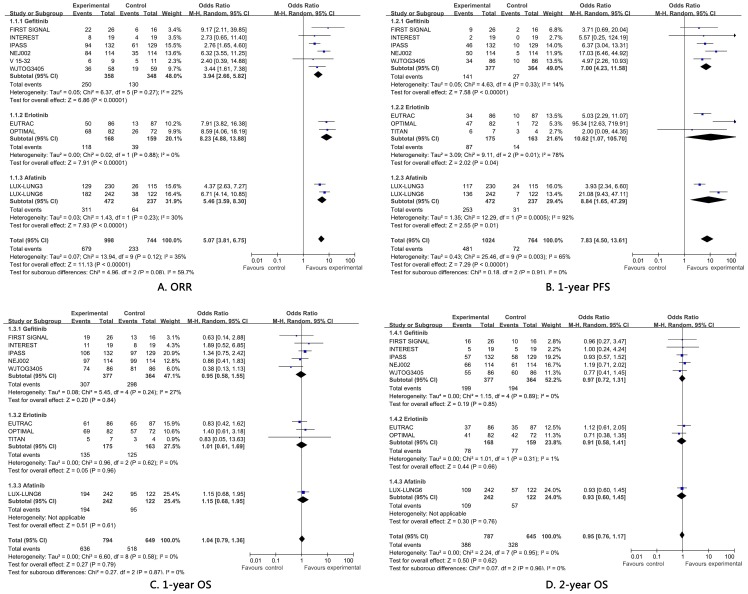
Direct meta-analyses of efficacy. a. objective response rate; b. 1-year progression free survival; c, 1-year overall survival; d, 2-year overall survival.

For mutated patients, the weighted pooled ORR and PFS of EGFR-TKIs were significant higher than standard chemotherapy. The pooled ORR was 66.6% (95%CI, 0.596 to 0.729) for TKIs versus 30.9% (95%CI, 0.245 to 0.381) for chemotherapy with an OR of 5.46 (95%CI, 3.59 to 8.30; *P*<0.00001). In terms of disease control, TKIs yielded 42.9% 1-year PFS (95%CI, 0.366 to 0.494) which was higher than that of chemotherapy 9.7% (95%CI, 0.058 to 0.158) with an OR of 7.83 (95%CI, 4.50 to 13.61; *P*<0.00001). Since OS data of ICOGEN for mutation population was unavailable, OS were not calculated for icotinib. The pooled 1-year and 2-year OS of TKIs was 79.2% (95%CI, 0.745 to 0.833) and 49.7% (95%CI, 0.432 to 0.563) respectively. On the other hand, the OS outcomes for chemotherapy were 78.9% (95%CI, 0.709 to 0.852) and 51.0% (95%CI, 0.432 to 0.563) for 2-year. Inconsistent with the results of ORR and PFS, OS data were similar between TKIs and chemotherapy (1-year: OR 1.04, 95%CI, 0.79 to 1.36, *P* = 0.79; 2-year: OR 0.95, 95%CI, 0.76 to 1.17, *P* = 0.62). Table 2 and Figure 2 presented all the pooled calculations and direct meta-analyses.

**Table 2:** Pooled Weighted Outcomes and Direct Meta-Analysis.

	TKIs (95% CI)	Chemotherapy (95% CI)	Odds Ratio (95% CI, P value)
**ORR**	66.6% (0.596, 0.729)	30.9% (0.245, 0.381)	5.46 (3.59, 8.30; P<0.00001)
**1-year PFS**	42.9%(0.366, 0.494)	9.7% (0.058, 0.158)	7.83 (4.50, 13.61; P<0.00001)
**1-year OS**	79.2% (0.745, 0.833)	78.9% (0.709, 0.852)	1.04 (0.79, 1.36; P = 0.79)
**2-year OS**	49.7% (0.432, 0.563)	51.0% (0.431, 0.589)	0.95 (0.76, 1.17; P = 0.62)

CI, confidence interval; ORR, objective response rate; PFS, progression free survival; OS, overall survival.

### Networks for Multiple Treatment Comparisons

**Figure 3:**
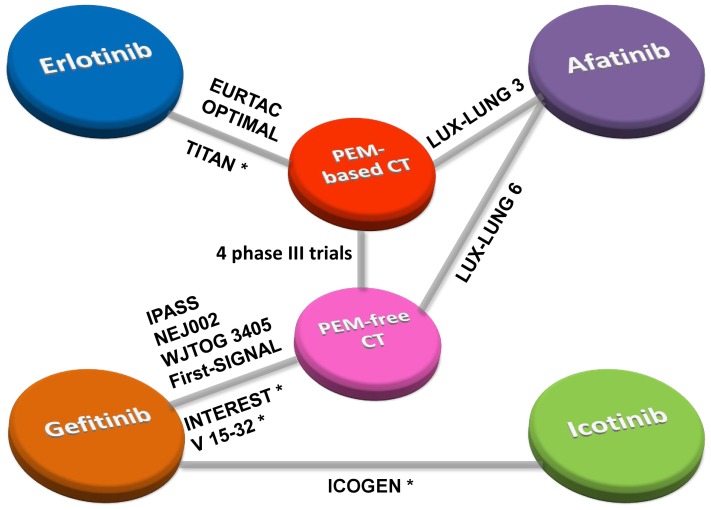
Network established for multiple treatment comparisons. Solid lines between drugs represented the existence of direct comparisons. PEM, pemetrexed; * Second-line studies.

We established two networks that included slightly different sets of studies, which considered sensitivity analyses as well (see Figure 3). Network 1 was the most extended one with all relevant evidence included. Network 2 considered studies investigating only first-line treatment.

### Network Meta-Analyses for Efficacy and Toxicities

Table 3 & 4 summarized the results of the multiple-treatments meta-analyses regarding ORR, 1-year PFS, 1-year OS and 2-year OS according to network 1 and 2, respectively. According to the results of network 1 and 2, elotinib, gefitinib, icotinib and afatinib shared equivalent efficacy in all outcome measures by showing no significant differences in ORs while all TKIs were better than chemotherapy (assessment of icotinib was not available neither in comparison of OS data nor in network 2). Coherence between direct and indirect comparisons based on networks was confirmed. We selected rash and diarrhea, which are the most common TKI-specific toxicities, as the representative of treatment-related toxicities. Patients who received afatinib experienced more severe diarrhea compared with the other three TKIs. In terms of rash, afatinib is significant severer than gefitinib while no other significant difference was observed among the rest comparisons. Afatinib and erlotinib had significant more grade 3 to 4 diarrhea or diarrhea compare with gefitinib and icotinib.

**Table 3:** Multiple treatment comparison for efficacy based on network 1.

**ORR**					
**PEM-based CT**	4.33 (2.09, 8.77)	0.60 (0.29, 1.26)	4.92 (1.79, 14.48)	2.45 (1.05, 5.81)	3.08 (0.71, 13.09)
0.23 (0.11, 0.48)	**Afatinib**	0.14 (0.07, 0.29)	1.14 (0.42, 3.30)	0.56 (0.25, 1.38)	0.72 (0.17, 3.14)
1.66 (0.80, 3.50)	7.09 (3.46, 14.70)	**PEM-free CT**	8.16 (3.87, 16.93)	4.00 (2.63, 6.50)	5.05 (1.46, 18.35)
0.20 (0.07, 0.56)	0.88 (0.30, 2.37)	0.12 (0.06, 0.26)	**Erlotinib**	0.50 (0.20, 1.16)	0.62 (0.14, 2.67)
0.41 (0.17, 0.96)	1.78 (0.73, 4.00)	0.25 (0.15, 0.38)	2.01 (0.86, 4.91)	**Gefitinib**	1.25 (0.40, 4.07)
0.32 (0.08, 1.41)	1.39 (0.32, 6.01)	0.20 (0.05, 0.68)	1.62 (0.37, 7.06)	0.80 (0.25, 2.53)	**Icotinib**
**1-year PFS**					
**PEM-based CT**	7.35 (1.46, 38.26)	0.67 (0.19, 2.16)	8.79 (1.28, 65.58)	5.34 (1.05, 27.63)	6.17 (0.36, 121.06)
0.14 (0.03, 0.68)	**Afatinib**	0.09 (0.02, 0.47)	1.19 (0.13, 11.91)	0.73 (0.10, 5.55)	0.86 (0.04, 20.93)
1.50 (0.46, 5.17)	11.05 (2.12, 59.61)	**PEM-free CT**	13.07 (2.95, 66.06)	7.94 (2.73, 26.22)	9.36 (0.78, 133.95)
0.11 (0.02, 0.78)	0.84 (0.08, 7.99)	0.08 (0.02, 0.34)	**Erlotinib**	0.60 (0.09, 4.30)	0.71 (0.04, 14.48)
0.19 (0.04, 0.95)	1.37 (0.18, 9.67)	0.13 (0.04, 0.37)	1.66 (0.23, 11.10)	**Gefitinib**	1.16 (0.12, 12.40)
0.16 (0.01, 2.76)	1.16 (0.05, 25.85)	0.11 (0.01, 1.27)	1.40 (0.07, 26.12)	0.86 (0.08, 8.22)	**Icotinib**
**1-year OS**					
**PEM-based CT**	0.85 (0.33, 1.98)	0.73 (0.49, 1.04)	0.75 (0.35, 1.56)	0.70 (0.36, 1.26)	
1.17 (0.50, 2.99)	**Afatinib**	0.87 (0.40, 1.99)	0.88 (0.32, 2.64)	0.83 (0.32, 2.19)	
1.36 (0.96, 2.04)	1.16 (0.50, 2.53)	**PEM-free CT**	1.03 (0.54, 1.94)	0.96 (0.57, 1.55)	
1.32 (0.64, 2.84)	1.14 (0.38, 3.11)	0.97 (0.52, 1.85)	**Erlotinib**	0.93 (0.41, 2.02)	
1.43 (0.79, 2.77)	1.21 (0.46, 3.10)	1.04 (0.64, 1.75)	1.07 (0.49, 2.46)	**Gefitinib**	
**2-year OS**					
**PEM-based CT**	0.89 (0.27, 2.92)	0.95 (0.55, 1.68)	0.84 (0.32, 2.33)	0.90 (0.42, 2.02)	
1.13 (0.34, 3.73)	**Afatinib**	1.07 (0.38, 3.15)	0.95 (0.26, 3.65)	1.02 (0.32, 3.46)	
1.06 (0.59, 1.82)	0.94 (0.32, 2.61)	**PEM-free CT**	0.90 (0.39, 2.06)	0.95 (0.55, 1.67)	
1.19 (0.43, 3.16)	1.06 (0.27, 3.88)	1.11 (0.49, 2.57)	**Erlotinib**	1.07 (0.39, 2.83)	
1.11 (0.49, 2.39)	0.98 (0.29, 3.09)	1.05 (0.60, 1.82)	0.93 (0.35, 2.54)	**Gefitinib**	
**Rash**					
**Afatinib**	0.02 (0.00, 0.06)	0.34 (0.05, 2.23)	0.19 (0.04, 0.96)	0.13 (0.01, 1.67)	
62.51 (15.60, 273.34)	**Chemotherapy**	21.19 (6.76, 72.96)	11.88 (5.58, 26.85)	8.05 (1.05, 67.83)	
2.92 (0.45, 18.80)	0.05 (0.01, 0.15)	**Erlotinib**	0.56 (0.13, 2.33)	0.38 (0.03, 4.33)	
5.24 (1.04, 26.81)	0.08 (0.04, 0.18)	1.78 (0.43, 7.64)	**Gefitinib**	0.67 (0.10, 4.72)	
7.84 (0.60, 96.53)	0.12 (0.01, 0.95)	2.64 (0.23, 28.64)	1.48 (0.21, 10.20)	**Icotinib**	
**Grade 3–4 Rash**					
**Afatinib**	0.00 (0.00, 0.00)	0.00 (0.00, 4E7)	0.00 (0.00, 0.03)	0.00 (0.00, 0.04)	
9E7 (202.47, 2E20)	**Chemotherapy**	5E5 (27.94, 1E11)	5.61 (1.80, 20.69)	5.95 (0.17, 214.81)	
5787.65 (0.00, 8E17)	0.00 (0.00, 0.04)	**Erlotinib**	0.00 (0.00, 0.21)	0.00 (0.00, 0.57)	
2E7 (34.20, 4E19)	0.18 (0.05, 0.55)	8714.47 (4.73, 2E10)	**Gefitinib**	1.05 (0.04, 28.74)	
2E7 (24.79, 6E19)	0.17 (0.00, 6.02)	8539.00 (1.76, 4E10)	0.96 (0.03, 28.05)	**Icotinib**	
**Diarrhea**					
**Afatinib**	0.01 (0.00, 0.03)	0.08 (0.02, 0.32)	0.03 (0.01, 0.10)	0.02 (0.00, 0.11)	
85.71 (31.42, 243.89)	**Chemotherapy**	6.98 (2.96, 17.89)	2.58 (1.47, 4.99)	1.55 (0.38, 7.08)	
12.32 (3.15, 46.68)	0.14 (0.06, 0.34)	**Erlotinib**	0.37 (0.13, 1.13)	0.22 (0.04, 1.32)	
32.97 (10.01, 101.94)	0.39 (0.20, 0.68)	2.69 (0.89, 7.77)	**Gefitinib**	0.59 (0.16, 2.25)	
56.36 (8.85, 314.64)	0.65 (0.14, 2.66)	4.57 (0.76, 24.16)	1.69 (0.45, 6.31)	**Icotinib**	
**Grade 3–4 Diarrhea**					
**Afatinib**	0.00 (0.00, 0.02)	3.35 (0.00, 2E14)	0.00 (0.00, 0.03)	0.00 (0.00, 0.01)	
1E7 (56.92, 3E11)	**Chemotherapy**	2E8 (30.20, 6)	1.49 (0.56, 7.69)	0.17 (0.00, 6.80)	
0.30 (0.00, 3E6)	0.00 (0.00, 0.03)	**Erlotinib**	0.00 (0.00, 0.06)	0.00 (0.00, 0.01)	
8E6 (31.39, 2E11)	0.67 (0.13, 1.78)	1E8 (16.87, 3E20)	**Gefitinib**	0.11 (0.00, 2.56)	
1E8 (152.26, 6E12)	5.84 (0.15, 398.10)	1E9 (98.66, 321)	9.00 (0.39, 642.15)	**Icotinib**	

CT, chemotherapy (not specific); PEM, pemetrexed;

**Table 4:** Rank probabilities of each TKI for different outcomes based on network 1.

Drug	Rank 1	Rank 2	Rank 3	Rank 4	Rank 5	Rank 6	Drug	Rank 1	Rank 2	Rank 3	Rank 4	Rank 5	Rank 6
**ORR**							**1-year PFS**						
**PEM-based CT**	0	0	0.01	0.06	0.86	0.07	**PEM-based CT**	0	0	0.02	0.08	0.7	0.2
**Afatinib**	0.29	0.47	0.19	0.05	0	0	**Afatinib**	0.27	0.3	0.21	0.21	0.01	0
**PEM-free CT**	0	0	0	0	0.08	0.92	**PEM-free CT**	0	0	0	0.01	0.22	0.76
**Erlotinib**	0.51	0.32	0.13	0.03	0	0	**Erlotinib**	0.38	0.3	0.18	0.14	0.01	0
**Gefitinib**	0.01	0.04	0.37	0.57	0.01	0	**Gefitinib**	0.06	0.22	0.42	0.29	0.01	0
**Icotinib**	0.19	0.17	0.3	0.29	0.05	0.01	**Icotinib**	0.29	0.18	0.18	0.27	0.05	0.03
**1-year OS**							**2-year OS**						
**PEM-based CT**	0.5	0.36	0.11	0.02	0.01		**PEM-based CT**	0.29	0.24	0.18	0.17	0.11	
**Afatinib**	0.3	0.21	0.14	0.14	0.2		**Afatinib**	0.27	0.14	0.1	0.17	0.32	
**CT**	0	0.09	0.36	0.4	0.15		**CT**	0.08	0.28	0.39	0.21	0.04	
**Erlotinib**	0.14	0.2	0.19	0.18	0.3		**Erlotinib**	0.19	0.14	0.13	0.2	0.33	
**Gefitinib**	0.05	0.14	0.2	0.26	0.34		**Gefitinib**	0.16	0.2	0.2	0.24	0.2	
**Rash**							**Grade 3–4 Rash**						
**Afatinib**	0.86	0.11	0.02	0.01	0		**Afatinib**	0.82	0.17	0	0	0	
**CT**	0	0	0	0.02	0.98		**CT**	0	0	0	0.13	0.86	
**Erlotinib**	0.09	0.64	0.18	0.09	0		**Erlotinib**	0.18	0.81	0.02	0	0	
**Gefitinib**	0.01	0.13	0.62	0.25	0		**Gefitinib**	0	0	0.48	0.51	0	
**Icotinib**	0.04	0.12	0.18	0.63	0.02		**Icotinib**	0	0.02	0.49	0.36	0.13	
**Diarrhea**							**Grade 3–4 Diarrhea**						
**Afatinib**	1	0	0	0	0		**Afatinib**	0.43	0.57	0	0	0	
**CT**	0	0	0	0.24	0.76		**CT**	0	0	0.17	0.7	0.13	
**Erlotinib**	0	0.94	0.05	0.01	0		**Erlotinib**	0.57	0.43	0	0	0	
**Gefitinib**	0	0.02	0.81	0.17	0		**Gefitinib**	0	0	0.77	0.22	0.01	
**Icotinib**	0	0.03	0.14	0.58	0.24		**Icotinib**	0	0	0.07	0.08	0.86	

CT, chemotherapy (not specific); PEM, pemetrexed;

### Rank Probabilities

**Figure 4:**
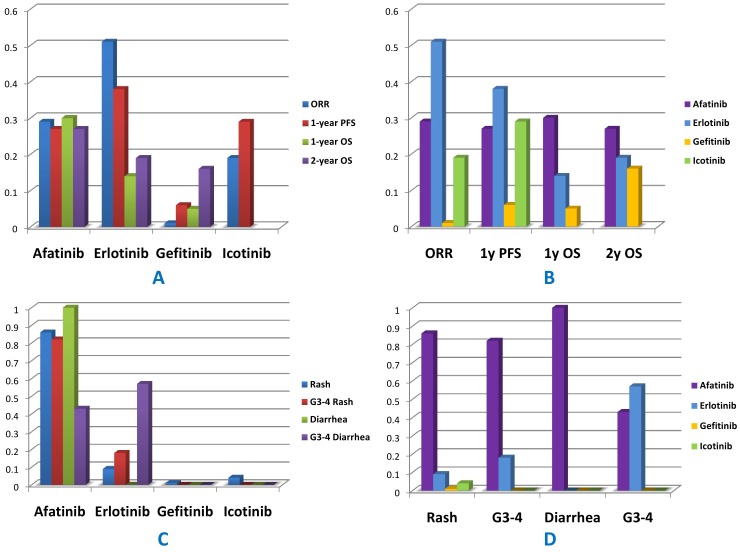
Distribution of probabilities of each agent being ranked the first place based on network 1. A & C were classified by drugs; B & D were classified by outcomes.

**Figure 5:**
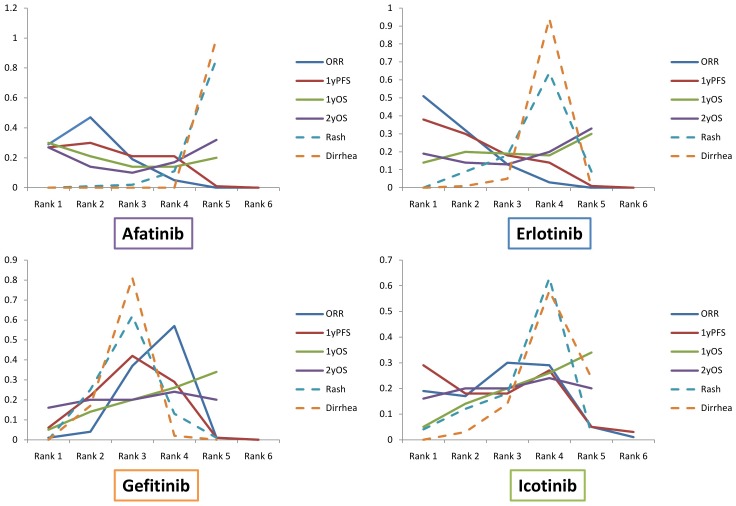
Distribution of probabilities of each agent being ranked at each of the possible positions.

Figure 4 was the ranking indicates the probability to be the best treatment, the second best, the third best, and so on, among all the treatment regimens. Agents with greater value in the histogram were associated with greater probabilities for better outcomes. Based on network 1, the cumulative probabilities of being the most efficacious treatments were (ORR, 1-year PFS, 1-year OS, 2-year OS): erlotinib (61%, 38%, 14%, 19%), gefitinib (1%, 6%, 5%, 16%), afatinib (29%, 27%, 30%, 27%) and icotinib (19%, 29%, NA, NA) (table 3). According to network 2 (1^st^-line studies only), the results were (ORR, 1-year PFS, 1-year OS, 2-year OS): erlotinib (61%, 61%, 15%, 19%), gefitinib (2%, 10%, 7%, 19%), afatinib (36%, 29%, 30%, 27%), whereas outcomes of icotinib were not assessable (table S1 in File S1). As visualized in the histogram in figure 4, we could see that erlotinib ranked best among all the TKIs in terms of ORR and 1-year PFS. Following erlotinib, icotinib and afatinib shared similar rankings with respect to ORR and 1-year PFS. Afatinib and erlotinib revealed superior OS rankings compared with the other two agents. Gefitinib was associated with relatively low probabilities to rank the first in efficacy outcomes. Figure 5 illustrated the distribution of probabilities of each treatment being ranked at each of the possible positions. Larger area under the curve at the left indicated better efficacy or tolerance. The detailed rank probabilities of each TKI for different outcomes were summarized in table 4 and table S2 in File S1.

## Discussion

Since a single trial usually compares only two or a few treatments (e.g. A vs. B, B vs. C), it is difficult to integrate information on the relative efficacy of all tested regimens for the same indication. Similarly, conventional direct meta-analysis also fails to measure the relative effect between diverse treatments as it only synthesizes trials with a same pair of comparators. Multiple-treatments comparison (MTC), or so called network meta-analysis, could compare a set of treatments for a specific disease simultaneously through a common comparator treatment [19]. For example, a trial compares treatment A with B while another compares B with C, a network consisting of A-B-C-(A) could be established by MTC, as well as an indirectly statistical relative effect on A versus C. When more treatments are involved (e.g. D, E, F), or evidences from certain pairs of treatments are sufficient to perform direct meta-analyses (e.g. two or more trials on A vs. B), the network gets improved and more approached to the reality. Moreover, Bayesian chain could help us rank these treatments to determine which one is most likely to be the best or the worst by measuring the corresponding probability [20]. For more rationale in detail of the MTC and Bayesian approach, please refer to http://drugis.org/gemtc. With several typical studies showing the good agreement between MTC and the real-world situation, MTC has been accepted as a reliable and an efficient method to compare different treatments [24], [31]. Therefore, it is highly suggested that considering all potentially relevant data when different treatments are indicated for a same disease [21].

This network meta-analysis was the first study using appropriate statistical methods to provide indirect comparison for the currently available EGFR-TKIs (erlotinib, gefitinib, afatinib and icotinib) in treating patients with advanced NSCLC who harbor EGFR mutations, based on all available information from phase III randomized trials. The superior efficacy of EGFR-TKIs for mutated population compared to chemotherapy has been substantially proved [32]. Nevertheless, direct head to head comparisons between these agents have not been well established. Despite some observational studies, only one phase II randomized, single-center, non-comparative phase II trial conducted by Kim et al. had evaluated the efficacy and safety of gefitinib and erlotinib as second-line therapy in highly selected advanced NSCLC patients according to clinical features [33]. Another registered phase II RCT that compares erlotinib to gefitinib in patients with exon21 mutation is on-going (NCT01024413 http://clinicaltrials.gov). Thus, no head-to-head comparison between these agents in EGFR-mutated populations has been available by far. These provided room and need for indirect and integrated comparisons. A recent pooled analysis of available studies was performed to evaluate clinical outcome in patients with EGFR-mutated NSCLC [34]. They pooled the overall median PFS and found it was 13.2 months with erlotinib, 9.8 months with gefitinib, and 5.9 months with chemotherapy. However, it did not prove whether the difference between erlotinib and gefitinib was statistically significant. Therefore, we sought to employ a novel indirect comparison method to draw more comprehensive conclusions on the substantial differences among these drugs. This would provide important information to facilitate both the phycisians and patients to choose from a group of agents that share similar mechanism.

For indirect methods, the underlying assumptions of the exchangeability of studies across the entire network should be examined carefully. We included only large phase III randomized trials with strict patient allocation and optimized balance between treatment and control arm to ensure the cross-study exchangeability. Besides, EGFR mutation status had been determined as the most remarkable predictor for EGFR-TKIs. In the current study, only patients with EGFR-mutation were included, which guarantee the homogeneity of study population. In addition, treatment-line might affect the efficacy and survival outcome of TKIs since a recent study suggested that chemotherapy might reduce EGFR mutation frequency [35]. Therefore, we established a modified network (network 2) to restrict the inclusion of first-line treatment only. In order to rule out the influence of the underlying bias in the retrospective biomarker analyses, we also established an additional network to exclude these studies (data not shown). Notably, we supplemented four studies that compared pemetrexed-based regimen with pemetrexed-free regimen in population with nonsquamous histology to optimize the network since pemetrexed has been proved to be superior to other third generation agents for nonsquamous carcinoma which is the dominant histological type of patients with EGFR mutation [36]. On the other hand, the equivalency of pemetrexed-free doublet regimens in terms of response rates and survival outcomes has been well established by a mile-stone RCT ECOG1594 and subsequent meta-analysis [37], [38]. Thus, we combined them as a single group in the networks. Through these efforts, the established model could be more concordant with real situation. Therefore, good coherence between direct and indirect comparison, as well as the tiny difference between random effects variance and inconsistency variance of each comparisons were observed, indicating that the consistency across the entire networks was guaranteed.

Firstly, we generated a set of pooled data according to the weight of each study which illustrated the current status of treatment with EGFR-TKIs. Based on such data, we could more intuitively show the true benefits which were given rise by EGFR-TKIs compared with traditional chemotherapy no matter in within-trials comparison or historical contrast, rather than merely reporting the OR/HR value. The superiority of EGFR-TKIs in ORR and PFS for EGFR-mutated patients indicated its specific efficacy in suppressing the tumor cells that were driven by the EGFR pathway. The failure to make a distinction between the OS outcomes of TKIs and chemotherapy could be explained by the influence of subsequent treatments [31]. Patients receiving chemotherapy as first-line treatments tended to take TKIs after progression whereas a smaller proportion of patients who previously received TKIs switched to chemotherapy probably due to the intolerance to toxicity [7], [8], [11]. As indicated by Zhou et al, patients who were able to receive both EGFR-TKI and chemotherapy regardless of the order had a significant longer median survival time compared with those received either TKI or chemotherapy only [39]. Therefore, the imbalance of subsequent treatments between TKI-group and chemo-group might mask the true benefits of EGFR-TKIs for overall survival.

Based on both network 1 and network 2, it was manifested that all currently available EGFR-TKIs were comparable in terms of ORR, PFS and OS (with only erlotinib, gefitinib and afatinib were compared for OS data) as treatments for EGFR mutated NSCLC patients. In a population perspective, no statistical differences between agents were observed. Rank probabilities provide us another perspective to review the position of certain treatment among all, especially when the relative values fail to reach statistical significance. The ranking could tell us which treatment would most likely be the best option, or whether one treatment is potentially better than another. In the current study, erlotinib had the greatest probability to rank the first among all the four TKIs regarding ORR and 1-year PFS while afatinib rank best in 1-year and 2-year OS. Icotinib shared similar rankings with afatinib respect to ORR and 1-year PFS. Gefitinib was associated with relatively low probabilities to rank the first but showed similar ranking compared with erlotinib in 2-year OS.

The trend of superiority of erlotinib versus gefitinib was in line with the previous pooled analysis [33]. Possible reasons for the trends were the differences in biological dose and mechanism between these agents. According to the phase I dose-escalation studies of these drugs, the reference doses of erlotinib (150 mg qd) and afatinib (40 mg qd) reached their maximum-tolerated doses (MTD) while gefitinib (250 mg qd) was administered at approximately one third of its MTD [40]–[42]. The MTD of icotinib was not reached in the dose-escalation study. Another index to evaluate the biological activity is the half-maximal inhibitory concentration (IC50) values, of which the lower value indicates better activity. The IC_50_ of erlotinib and icotinib for molecule level or cellular level was similar and significant smaller than that of gefitinib [43]. The biological activity was associated with the potential difference in tumor sensitivity of EGFR TKIs. The pharmacokinetic data was in good agreement with the efficacious rankings observed in the current study. On the other hand, afatinib is considered as a second-generation TKI that irreversibly inhibits EGFR-kinases. Some evidences showed its activity in treatment of patients with a secondary T790 mutation which accounts for approximately 50% cases of acquired resistance to EGFR-TKI treatments [17].In addition, it is a ‘pan-HER’ inhibitor that targets all ErbB receptor family (HER 1–4) [17]. Therefore, afatinib is not only active against EGFR mutations targeted by first-generation TKIs like erlotinib or gefitinib, but also against other signaling networks that were not sensitive to previous therapies. This specific underlying mechanism might be a reason for the satisfying outcomes of afatinib especially the long-term OS data.

Although presenting potentially better efficacy, erlotinib and afatinib were associated with significant higher toxicities compared with gefitinib and icotinib. Through reviewing the distribution of rank probabilities in figure 5, we could summarize that erlotinib and afatinib showed a high efficacy-high toxicity pattern whereas gefitinib and icotinib showed a medium efficacy-moderate toxicity pattern. Tolerance should not be ignored since a substantial proportion of patients might undergo discontinuation of treatment that related to intolerable adverse events [44]. Physicians are suggested to carefully weigh and balance the benefits and risks.

This is the first multiple treatment comparison for the currently available EGFR-TKIs in treating EGFR mutated advanced NSCLC patients based on evidences with good quality. The finding that efficacy was equivalent among these agents might help clinicians in making decisions. However, there existed several limitations. First, OS data in mutant population of ICOGEN were not available hence we could not evaluate the survival benefits of icotinib. In addition, comparisons in terms of OS were confounded by subsequent treatments. Second, the performance of icotinib in first-line setting was not available. Third, we could not assess some important molecular markers including T790M status in the population which might have effects on the efficacy of TKIs and cause bias. Fourth, the established networks lacked sufficient direct comparisons between TKIs. At last, we could not evaluate the adverse events in mutant population in the current study since no specific data from these patients were reported by the included trials. As we know, the overall data of adverse events might be influenced by the proportion of mutated patients. Therefore, future head-to-head randomized trials which would optimize the network and multiple treatment comparison based on individual patient data are warranted to further clarify these issues. Novel TKIs in the second- or third- generation such as canertinib, dacomitinib and CO-1686 were expected to be included.

In conclusion, this network meta-analysis indicated that erlotinib, gefitinib, afatinib and icotinib shared equivalent efficacy but presented different efficacy-toxicity pattern for EGFR-mutated patients according to current evidences. We suggested that physicians fully consider the efficacy-toxicity balance to select appropriate TKI for patients.

## Supporting Information

Checklist S1 **PRISMA checklist.**
(DOC)

File S1 **Table S1, Multiple treatment comparison for efficacy based on network 2 (1st-line studies only). Table S2, Rank probabilities of each TKI for different outcomes based on network 2 (1st-line studies only).**
(DOC)
