# Supplementary material for: Network Meta-Analysis of Erlotinib, Gefitinib, Afatinib and Icotinib in Patients with Advanced Non-Small-Cell Lung Cancer Harboring EGFR Mutations
Source: PLoS One. 2014 Feb 12;9(2):e85245. doi: 10.1371/journal.pone.0085245 (PMC3922700; doi:10.1371/journal.pone.0085245)
Supplement: File S1 — Table S1, Multiple treatment comparison for efficacy based on network 2 (1st-line studies only). Table S2, Rank probabilities of each TKI for different outcomes based on network 2 (1st-line studies only). (DOC) [file pone.0085245.s002.doc]

**Table S1 Multiple treatment comparison for efficacy based on network 2 (1^st^-line studies only)**

| **ORR** | | | | |
| --- | --- | --- | --- | --- |
| **Pemetrexed-based CT** | 4.24 (1.85, 10.15) | 0.59 (0.25, 1.40) | 5.04 (1.53, 16.17) | 2.53 (0.93, 7.49) |
| 0.24 (0.10, 0.54) | **Afatinib** | 0.14 (0.06, 0.32) | 1.19 (0.37, 3.79) | 0.59 (0.22, 1.73) |
| 1.70 (0.71, 3.98) | 7.16 (3.09, 16.91) | **Chemotherapy** | 8.59 (3.80, 19.06) | 4.25 (2.50, 8.10) |
| 0.20 (0.06, 0.65) | 0.84 (0.26, 2.72) | 0.12 (0.05, 0.26) | **Erlotinib** | 0.51 (0.19, 1.41) |
| 0.40 (0.13, 1.07) | 1.69 (0.58, 4.54) | 0.24 (0.12, 0.40) | 1.98 (0.71, 5.23) | **Gefitinib** |
| **1-year PFS** | | | | |
| **Pemetrexed-based CT** | 7.30 (1.27, 45.22) | 0.67 (0.19, 2.36) | 11.29 (1.47, 126.74) | 4.98 (0.88, 28.95) |
| 0.14 (0.02, 0.79) | **Afatinib** | 0.09 (0.02, 0.54) | 1.55 (0.14, 23.97) | 0.69 (0.08, 5.77) |
| 1.49 (0.42, 5.39) | 10.96 (1.87, 66.38) | **Chemotherapy** | 17.00 (3.26, 120.64) | 7.45 (2.23, 26.27) |
| 0.09 (0.01, 0.68) | 0.64 (0.04, 7.24) | 0.06 (0.01, 0.31) | **Erlotinib** | 0.44 (0.04, 3.25) |
| 0.20 (0.03, 1.13) | 1.46 (0.17, 13.28) | 0.13 (0.04, 0.45) | 2.30 (0.31, 23.62) | **Gefitinib** |
| **1-year OS** | | | | |
| **Pemetrexed-based CT** | 0.85 (0.31, 2.11) | 0.73 (0.47, 1.05) | 0.75 (0.33, 1.69) | 0.72 (0.35, 1.36) |
| 1.17 (0.47, 3.27) | **Afatinib** | 0.85 (0.37, 2.12) | 0.88 (0.29, 2.81) | 0.84 (0.31, 2.38) |
| 1.37 (0.95, 2.14) | 1.18 (0.47, 2.70) | **Chemotherapy** | 1.03 (0.53, 2.12) | 0.99 (0.57, 1.66) |
| 1.33 (0.59, 3.06) | 1.14 (0.36, 3.40) | 0.97 (0.47, 1.90) | **Erlotinib** | 0.96 (0.38, 2.25) |
| 1.39 (0.74, 2.85) | 1.18 (0.42, 3.22) | 1.01 (0.60, 1.76) | 1.04 (0.44, 2.66) | **Gefitinib** |
| **2-year OS** | | | | |
| **Pemetrexed-based CT** | 0.88 (0.25, 3.07) | 0.94 (0.54, 1.71) | 0.85 (0.32, 2.38) | 0.90 (0.41, 2.10) |
| 1.14 (0.33, 3.94) | **Afatinib** | 1.08 (0.36, 3.30) | 0.97 (0.24, 3.90) | 1.04 (0.29, 3.67) |
| 1.06 (0.58, 1.84) | 0.92 (0.30, 2.80) | **Chemotherapy** | 0.89 (0.39, 2.09) | 0.96 (0.53, 1.76) |
| 1.18 (0.42, 3.15) | 1.03 (0.26, 4.10) | 1.12 (0.48, 2.55) | **Erlotinib** | 1.08 (0.38, 2.99) |
| 1.11 (0.48, 2.44) | 0.96 (0.27, 3.45) | 1.04 (0.57, 1.90) | 0.93 (0.33, 2.65) | **Gefitinib** |

**Table S2 Rank probabilities of each TKI for different outcomes based on network 2 (1^st^-line studies only)**

| **Drug** | **Rank 1** | **Rank 2** | **Rank 3** | **Rank 4** | **Rank 5** |  | **Drug** | **Rank 1** | **Rank 2** | **Rank 3** | **Rank 4** | **Rank 5** |  |
| --- | --- | --- | --- | --- | --- | --- | --- | --- | --- | --- | --- | --- | --- |
| **ORR** | | | | | | | **1-year PFS** | | | | | | |
| A-based CT | 0.00 | 0.01 | 0.03 | 0.88 | 0.09 |  | A-based CT | 0.00 | 0.01 | 0.04 | 0.74 | 0.21 |  |
| Afatinib | 0.36 | 0.52 | 0.11 | 0.00 | 0.00 |  | Afatinib | 0.29 | 0.41 | 0.28 | 0.01 | 0.01 |  |
| Chemotherapy | 0.00 | 0.00 | 0.00 | 0.09 | 0.91 |  | Chemotherapy | 0.00 | 0.00 | 0.01 | 0.21 | 0.78 |  |
| Erlotinib | 0.61 | 0.33 | 0.06 | 0.01 | 0.00 |  | Erlotinib | 0.61 | 0.26 | 0.12 | 0.01 | 0.00 |  |
| Gefitinib | 0.02 | 0.14 | 0.81 | 0.03 | 0.00 |  | Gefitinib | 0.10 | 0.32 | 0.55 | 0.03 | 0.00 |  |
| **1-year OS** | | | | | | | **2-year OS** | | | | | | |
| A-based CT | 0.47 | 0.36 | 0.12 | 0.04 | 0.01 |  | A-based CT | 0.28 | 0.25 | 0.19 | 0.17 | 0.11 |  |
| Afatinib | 0.30 | 0.21 | 0.15 | 0.13 | 0.21 |  | Afatinib | 0.27 | 0.13 | 0.10 | 0.16 | 0.33 |  |
| Chemotherapy | 0.00 | 0.09 | 0.33 | 0.42 | 0.16 |  | Chemotherapy | 0.08 | 0.29 | 0.38 | 0.21 | 0.05 |  |
| Erlotinib | 0.15 | 0.18 | 0.18 | 0.18 | 0.31 |  | Erlotinib | 0.19 | 0.14 | 0.13 | 0.22 | 0.32 |  |
| Gefitinib | 0.07 | 0.16 | 0.22 | 0.23 | 0.32 |  | Gefitinib | 0.19 | 0.19 | 0.20 | 0.24 | 0.20 |  |

CT, chemotherapy (not specific); A, pemetrexed;
